# A Quantile Regression Analysis of Factors Associated with First-Time Maternal Fatigue in Korea

**DOI:** 10.3390/ijerph19010215

**Published:** 2021-12-25

**Authors:** Jeongok Park, Chang Gi Park, Kyoungjin Lee

**Affiliations:** 1 Mo-Im Kim Nursing Research Institute, College of Nursing, Yonsei University, Seoul 03722, Korea; jopark02@yuhs.ac; 2 Department of Population Health Nursing Science, College of Nursing, University of Illinois, Chicago, IL 60607, USA; parkcg@uic.edu; 3 College of Nursing and Brain Korea 21 FOUR Project, Younsei University, Seoul 03722, Korea

**Keywords:** fatigue, mothers, sleep quality, parenting stress, child behavior, children, sleep problems, nursing

## Abstract

The aim of this cross-sectional study was to identify the factors associated with different percentiles of first-time maternal fatigue. A total of 123 first-time healthy mothers aged 18 years or older participated through an online survey. The fatigue was measured by the Korean version of the fatigue severity scale. Main variables were constructed based on the integrated fatigue model, which included mothers’ sleep quality, parenting stress, the amount of free time mothers have, the number of the child’s night wakings, general characteristics including socioeconomic status, and working status. Quantile regression was used to analyze the associated factors according to the fatigue level of first-time mothers with a young child. The mean age of the mothers and children were 32.11 years and 20.81 months, respectively. Mean fatigue score was 6.16 among the 75% quantile with high fatigue score. Lack of adequate free time in mothers, advanced maternal age, being a housewife, having a moderate income, and frequent night wakings of their child significantly increased fatigue among mothers in the third quantile of fatigue. To reduce fatigue, healthcare providers should focus on exploring ways to reduce maternal sleep disturbance and improve maternal sleep quality.

## 1. Introduction

The symptom of fatigue is a common problem in women after childbirth and the prevalence of fatigue after childbirth has been reported to range from 55.0% to 69.0% [1,2]. Fatigue is defined as a sign and symptom of subjective feelings of tiredness caused by changes in aspects as biological and psychosocial factors [3]. Maternal fatigue is not a life-threatening condition; however, it negatively affects both the mother’s physical [2] and emotional health [4] as well as the child’s development [5].

In the literature, quality of sleep, parenting stress, and social support have been reported as key factors associated with maternal fatigue [5,6,7,8]. The quality of maternal sleep is affected by the child’s sleep pattern [9,10]. Compared to mothers whose infants were solitary sleepers, persistent co-sleeping with their child was associated with sleep disruptions in mothers but not in infants [9]. Especially in Korea, co-sleeping is a natural part of parenting and most Korean parents (94.5%) sleep with their children in the same room or their bed [11]. In addition, the sleeping patterns of infants is different from that of children aged 2 to 3 years old. Infants are characterized by frequent waking at night without integrated sleep owing to immature 24-h circadian rhythms [12]; however, sleep onset-latency or insomnia is common in children aged 2–3 years [13]. Whether an infant or young child, having a sleep problem in a child adversely affects maternal health [14]. Maternal fatigue is also associated with parenting stress [5], which is defined as the unpleasant experience arising from the interaction between parents and their children in the process of parenting [15]. Cooklin et al. (2012) identified the relationship between parenting stress, parenting competence, and fatigue in mothers and fathers and found that higher parental fatigue was significantly associated with higher parenting stress and lower parenting competency, measured in terms of parenting self-efficacy and satisfaction. Higher fatigue was associated with recognizing one’s child with demanding disposition, exhaustion with parenting, and lack of satisfaction and parenting ability. Social support, such as family support, is another significant factor of maternal fatigue [3,5,16]. Family support is needed by mothers in postpartum to reduce fatigue and it is an essential resource that allows resting and recuperating [16]. Cooklin et al. (2012) found that low satisfaction with existing family support, such as assistance with household tasks, was associated with higher fatigue [5]. In addition, Piper et al. (1987) explained that changes in the form of activities and rest could play an important role in the prevention and mitigation of fatigue [3].

Maternal fatigue is higher in first-time mothers than in mothers with prior parenting experience [4,17]. First-time mothers were more stressed and spent more time rearing their child because of a lack of experience with childcare compared with mothers with prior parenting experience [17]. Specifically, the main stressor for first-time mothers was related to direct care for their child, such as neonatal bathing and feeding [4].

Some studies have reported that maternal fatigue is not only limited to a short period after childbirth but also persists from six months to several years after childbirth [18,19,20]. For example, Giallo et al. (2015) found that a moderate to high level of maternal fatigue had been maintained without any change for several months. Another study found that mothers were more fatigued and had a lower level of energy at 14 to 19 months after childbirth compared with mothers in early postpartum [20]. Furthermore, maternal fatigue was more severe during the early childhood period (from childbirth to three years old), where the energy demands of parenthood are considerably higher [21]. Although maternal fatigue has been reported in mothers rearing young children as well as infants, most studies on maternal fatigue have focused on short-term periods after childbirth to as much as six months after childbirth, and little is known about the fatigue of mothers rearing young children.

**Figure 1:**
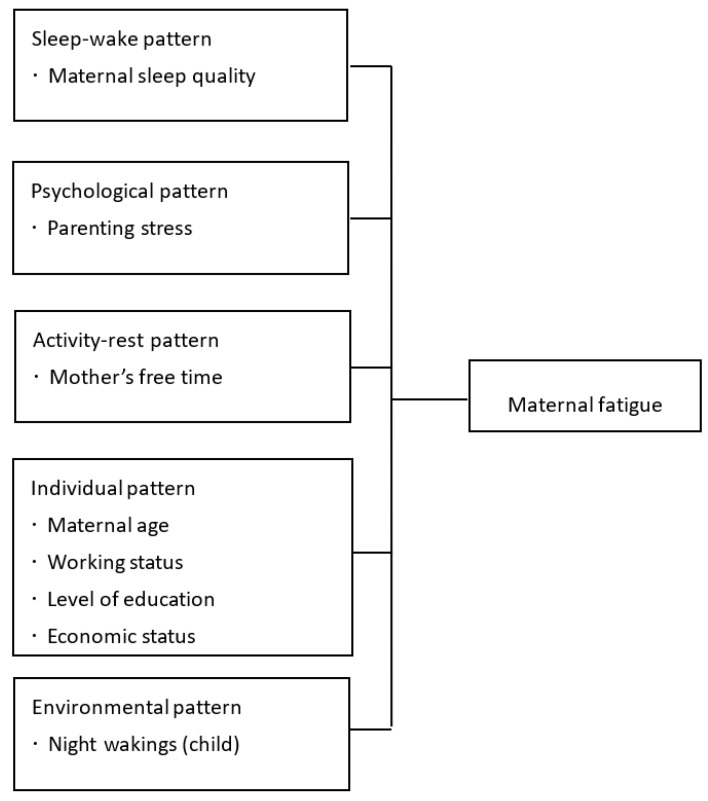
Conceptual Framework of the Study.

The conceptual framework of this study (Figure 1) was developed based on Piper’s integrated fatigue model [3]. This model accounts for sleep-wake, psychological, activity-rest, individual, and environmental patterns. In the current study, sleep-wake pattern refers to mothers’ sleep quality; psychological pattern, parenting stress; activity-rest pattern, the amount of free time mothers have; individual pattern, general characteristics, socioeconomic status, and working status; and environmental pattern, the number of child’s night wakings.

Most of the estimation strategies in previous studies related to maternal fatigue were usually analyzed with the ordinary least squares (OLS) method, which is a common statistical method used by researchers to investigate relationships between variables [22].

This method typically assumes that associations between the independent and dependent variables are the same at all levels and it can confirm effects on the medians of dependent variables [22]. On the other hand, quantile regression analysis is a method of estimating functional relationships between variables in all the parts of a probability distribution [23]; one advantage is that this analysis can be performed for different percentiles as well as the mean of the distribution of dependent variables [24]. Quantile regression is an ideal method for estimating the conditional quantiles of a response variable distribution in a linear model, which provides a more complete view of possible causal relationships between variables.

In summary, it is known that maternal fatigue is related to maternal sleep quality, parenting stress, mother’s free time, family support, maternal age, social-economic status, and night wakings in their child. However, previous studies have focused on the fatigue of mothers, regardless of whether they are primipara or multipara [25]. In addition, little is known as to which factors are related to the fatigue level of mothers.

The purpose of this study was to identify the factors developed based on the integrated fatigue model mentioned above associated with different percentiles of first-time maternal fatigue using quantile regression.

## 2. Materials and Methods

### 2.1. Study Design

A cross-sectional design was used in this study.

### 2.2. Ethical Consideration

The Institutional Review Board of Yonsei University in Korea (No. Y-2018-0044) approved this study.

### 2.3. Sample Size

The sample size was calculated using the G*power program before starting the study. We obtained a minimum sample size of 109 using linear multiple regression with effect size 0.15, significance level 0.05, power 0.8, and eight predictors. Considering the attrition rate of 15%, the sample size was calculated 128. Thus, the sample size of 123 was appropriate for having appropriate power.

### 2.4. Participants

The following questions were used to screen for eligibility. “How many children do you have?” “How old is your child?” “What year were you born?” “Did you receive treatment for more than 6 months after being diagnosed with a specific disease?” A total of 123 women were included. The inclusion criteria were; women who (1) were first-time mothers rearing a child three years old or younger, (2) were aged 18 years old or older, and (3) did not have a current illness or were not taking medications.

### 2.5. Data Collection

The data were collected between 30 May and 19 June in 2018 through an online survey from Korean online popular communities. First, a research assistant sent an e-mail regarding the research topic to potential participants in the survey community. Second, if potential participants were interested in the research topic after reading a summarized introduction of this study, they were screened for eligibility using questionnaires. Third, upon meeting the inclusion criteria, they were guided to click and read subsequent pages for a detailed description of the study. Then, participants read the detailed description of this study and were asked, “Do you agree to participate in this study?” If they clicked the button, it was considered as their agreement to participate.

### 2.6. Variables

#### 2.6.1. Fatigue

Maternal fatigue was assessed using the Korean version of the fatigue severity scale [26]. The fatigue severity scale was originally developed by Krupp et al. [27] and is a self-reported measure consisting of 9 items that assess the degree of fatigue during the previous week. Each question is rated on a 7-point Likert scale, with high scores indicating a high degree of fatigue. The Cronbach’s alpha of the original version was 0.88 and that of the Korean version was 0.87. In the current study, it was 0.92.

#### 2.6.2. Sleep Quality

Maternal sleep quality was measured using the Korean version of the Pittsburgh Sleep Quality Index [28,29]. The Pittsburgh sleep quality index is a self-rated questionnaire that assesses sleep quality and sleep disturbances over a one-month time interval. The scores range from 0 to 21 and higher scores indicate lower sleep quality. The Cronbach’s alpha of the Korean version was 0.84 and in this study it was 0.76.

#### 2.6.3. Parenting Stress

Parenting stress was measured using the Korean version of the parenting stress index short-form [30]. The parenting stress index short-form was developed by Abidin in 1995 [31] and designed as a screening and diagnostic assessment instrument for identifying problems in terms of parent and child systems that are under stress and in which deviant development of the child is likely to take place or dysfunctional parenting is likely to occur [31]. The parenting stress index short-form contains 36 of the original 120 items in the parenting stress index. The total scores are the sums of scores and high scores indicate high parenting stress. The Cronbach’s alpha of the Korean version ranged from 0.76 to 0.91. In this study, it was 0.76.

#### 2.6.4. Free Time of Mother

The amount of mothers free time was measured by the question, “Did you have time during the day to enjoy physical and mental rest and relaxation, such as exercise, reading a book, and having tea time, that was only for yourself by getting help related to childcare or housework from others (e.g., husbands, parents, babysitters, etc.)?” Mothers who answered “yes” were asked the following question, “In the last month, how many hours of free time did you take in a day on average?” The answer was in terms of minutes and for mothers who answered “no,” the response was coded as 0 min.

#### 2.6.5. General Characteristics

The general characteristics of participants that were assessed included age (year), level of education (high school graduate, college graduate, graduate school or higher), working status (yes, no) and economic status (low, middle, high).

#### 2.6.6. The Number of Child’s Night Wakings

The number of the child’s night wakings was assessed by the question “In the last month, how many times did your child wake up at night (from 7:00 p.m. to 7:00 a.m.) on average?” The possible responses were “zero,” “once,” “twice,” or “more than thrice”.

#### 2.6.7. Data Analysis

The quantile regression method was used for identifying factors related to being a first-time mother with a young child according to the level of fatigue, especially in the 75% quantile with high fatigue scores. This study also used OLS to compare the results with those of quantile regression. Quantile regression analysis provides greater flexibility compared to other regression methods in identifying differing relationships at different parts of the distribution of a dependent variable [24].

Descriptive statistics such as means, standard deviations, or percentages were used to describe the participants’ characteristics. To examine multicollinearity between the explanatory variables, variance inflation factor and condition index were examined. Consequently, the variable of child age was excluded from the main analysis because it had a condition index value of 35.03 and was highly correlated with the number of the child’s night wakings. All statistical analysis was performed using SAS software (SAS Institute, Inc., Cary, NC, USA), Version 9.4 of the SAS System [32]. 

## 3. Results

### 3.1. Maternal and Child Characteristics

General characteristics of mothers and children are presented in Table 1.

#### 3.1.1. General Characteristics of Mothers

A total of 123 mothers with young children were included in this study. Their mean fatigue score was 4.84 (SD = 1.08), ranging from 1.78 to 6.89, and the mean fatigue scores in the 25%, 50%, and 75% quantiles were 3.33 (SD = 0.72), 4.92 (SD = 0.46), and 6.16 (SD = 0.31), respectively. The mean maternal sleep quality score was 8.75 (SD = 3.32), ranging from 2 to 17. The mean parenting stress score was 93.81 (SD = 21.13) and about 20% of the participants were categorized as having very high parenting stress. The mean free time of mothers was 85.98 (SD = 115.32) minutes a day. The mean age of mothers was 32.11 (SD = 3.80) years, with a ranging from 22 to 42 years old. By family type, the majority (91.9%) were nuclear families. Approximately 59% of the participants had a job and about three-fourth of them had graduated from college. Furthermore, 54.5% of the participants (*n* = 67) had a monthly household income less than 2700 USD, and only 6.5% reported a monthly households income of 5000 USD or more.

#### 3.1.2. General Characteristics of Children

The mean age of the children was 20.81 (SD = 9.54) months, ranging from three to 36 months. Approximately half of the children woke up more than twice per night.

### 3.2. Factors Associated with Maternal Fatigue

The results of the OLS and quantile regression analyses are presented in Table 2. The results of the OLS analysis showed that maternal sleep quality (β = 0.14, SE = 0.03, *p* < 0.0001) and the group with the highest parenting stress (β = 0.71, SE = 0.24, *p* = 0.004) were significantly associated with maternal fatigue.

Similarly, maternal sleep quality (25%: β = 0.21, SE = 0.03, *p* < 0.0001; 50%: β = 0.13, SE = 0.03, *p* < 0.0001; 75%: β = 0.05, SE = 0.02, *p* = 0.005) and parenting stress (25%: β = 0.70, SE = 0.27, *p* = 0.011; 50%: β = 0.73, SE = 0.22, *p* = 0.001; 75%: β = 0.59, SE = 0.15, *p* = 0.0002) were significantly associated with maternal fatigue in the quantile regressions.

Furthermore, the maternal fatigue level of the 75% quantile was significantly associated with amount of mother’s free time (β = −0.002, SE = 0.001, *p* = 0.004), maternal age (β = 0.04, SE = 0.02, *p* = 0.017), working mothers (β = −0.36, SE = 0.12, *p* = 0.003), the group with moderate monthly income (β = 0.27, SE = 0.12, *p* = 0.025), group with high monthly income (β = −0.84, SE = 0.23, *p* = 0.0004), and three or more instances of child’s night wakings (β = 0.43, SE = 0.18, *p* = 0.022). These were not significant in the OLS analysis.

## 4. Discussion

The purpose of this study was to identify factors associated with maternal fatigue based on Piper’s integrated fatigue model. The major findings of this study are as follows: (1) sleep-wake and psychological patterns such as maternal sleep quality and parenting stress were significantly associated with maternal fatigue; (2) activity-rest, individual, and environmental patterns such as free time, mother’s current age, working status, economic status, and number of the child’s night wakings were associated with a high level of fatigue.

The present results showed that maternal sleep quality and parenting stress were significantly associated with all quantiles of maternal fatigue, which is consistent with previous studies [5,8,33]. Regarding maternal sleep quality, Gay et al. (2004) found a significant association between increased fatigue and sleep disturbances measured by wrist actigraphy and questionnaires among 72 couples in the postpartum period [33]. Similarly, Rychnovsky and Hunter (2009) found that the fatigue of postpartum women as measured by questionnaires was associated with disturbed sleep in terms of frequent awakenings, time spent awake, sleep depth, and difficulty falling asleep [8]. Cooklin et al. (2012) reported sleep disruption as a key factor contributing to fatigue and found that maternal fatigue was more severe than paternal fatigue [5]. Sleep interventions considering the sleep patterns of mothers and their children are necessary to improve maternal sleep quality because they spend a significant amount of time together during the day and night.

Previous studies have reported parenting stress as a significant factor associated with maternal fatigue [6,21] and the level of fatigue remained high from after giving birth to more than a year after childbirth [20] In most cultures, women provide more childcare than men do. In particular, Korea has the lowest male participation time in housework among OECD countries [34]. The additional roles of mother and wife that accompany marriage are burdensome for women in Korea [35]. Thus as the primary caregivers, mothers rearing children often experience a high level of parenting stress [15,36]. The level of fatigue remains high from after giving birth to more than a year after childbirth [20,37]. Moreover, the burden and parenting stress of childcare in Korean married women, especially first-time mothers, were much higher than that of women in other countries [38]; Thus, the imbalance in the distribution of gender roles may result in emotional problems [35]. The burden and fatigue of new roles are significant for first-time mothers. Therefore, intervention is needed for mothers with severe fatigue by identifying the factors affecting fatigue.

A previous study found that the degree of parenting stress and factors influencing mothers’ stress differed by the developmental stages of the child [39]. For instance, intimacy with a partner was mainly related to mothers’ stress in the early childbirth period and the child’s temperament was mainly related to mothers’ stress in the first two or three years when the child’s personality is formed. To reduce maternal fatigue, healthcare providers can offer mothers information about their child’s developmental stage and conduct interventions to reinforce parental competence.

In the current study, having free time was associated with a low level of fatigue. Similarly, Musick, Meier, and Flood (2016) found that the quantity and quality of free time away from parenting might affect parents’ stress, fatigue, and feelings defined as well-being indicators [40]. In detail, free time was measured as hours of free time in the prior 24-h period and the results showed that experiencing free time was associated with lower maternal stress and fatigue.

With regard to individual patterns including age and working and economic status, the findings of previous studies have been inconsistent. The current study showed that being older significantly increased maternal fatigue among mothers with a high level of fatigue. This finding is meaningful in Korea because Korean women’s mean age at first childbirth had increased from 26.7 years in 1996 to 31.2 years in 2015 [41]. However, the findings of previous studies on the association between mother’s age and fatigue are inconsistent. Song et al. (2007) and Taylor and Johnson (2013) found that older maternal age was associated with a higher level of fatigue [16,25]; however, Giallo et al. (2015) found that older mothers experienced less fatigue than did younger mothers at early postpartum [18]. However, these results should be interpreted with caution because those studies included both primiparous and multiparous mothers during a short period after childbirth (up to six months).

Regarding working status, having a job decreased fatigue among mothers in the third quantile in the current study. In similar, Kılıç & Eryılmaz (2011) found that unemployed mothers were more fatigued in the postpartum period [42]; however, Farag & Hassan (2019) found that 250 postpartum primiparous women were more fatigued compared with full-time housewives [43]. In the other studies, there were no statistically significant differences between fatigue level and maternal working status [44]. Comparing the finding of the current study with them of previous studies does not seem to be appropriate because of different participants’ characteristics of the studies. The participants of previous studies were at the postpartum period from six weeks to three months after childbirth, while the current study included mothers from three to 36 months after childbirth. Because most developed countries, including those of the participants in the previous studies mentioned above and Korea, provide women with three months of maternity leave, it is unclear whether they actually work at the workplace during the postpartum period because they might be on maternity leave.

In the literature, it was reported that socioeconomic status was negatively associated with the level of fatigue in mothers [18,45] which was consistent with the current study. Giallo et al. (2015) found that a low level of socioeconomic status was associated with high levels of fatigue in the first seven months postpartum [18]. Doering and Durfor (2011) conducted qualitative research among low-income urban mothers in the first six months postpartum and found that mothers rearing children were burdened with performing diverse roles caused by economic and situational difficulties, which resulted in the accumulation of sleep deprivation and consequently contributed to fatigue [45].

Lastly, one of the main factors contributing to fatigue was having children with a disturbed sleep pattern, which may lead to insufficient sleep in mothers. Specifically, three or more night wakings among children was significantly associated with increased maternal fatigue in only the third fatigue quantile. This finding was consistent with the study by Gay et al. (2004), who found that mothers who were disturbed by the sleep pattern of their child at night were more likely to have high fatigue [33]. This finding indicates that developing interventions that consider the child’s sleep pattern is necessary. Maintaining a good sleep environment is also important for improving the sleep quality of mothers in order to decrease maternal fatigue. Bed sharing may include benefits such as an improved parent-child relationship and low separation anxiety in the child; however, it can affect maternal sleep quality [33] and increase the risk of sudden infant death syndrome (SIDS) [46]. In Korea, most parents (94.5%) sleep with their children in the same room or their bed [11]. Although the American Academy of Pediatrics officially discourages bed sharing, the proportion of bed sharing between mother and child increased in the US from 5.55% to 12.8% between 1993 and 2000 [47]. One workaround could be allowing the mother and child to sleep together in the same room but using separate beds. A risky sleep environment (e.g., not using a firm mattress) and improper posture, which can interfere with breathing, are possible causes of SIDS. Using separate beds in the same room can help maintain a safe sleep environment as well as improve the interaction between mother and child.

This study has several limitations. First, this study has some limitations in the sampling method and representativeness of the participants. A convenient sampling method was used for the recruitment of participants in this study and thus there are limitations to the generalization of research findings. Because the participants of this study were recruited through famous online communities where many Korean mothers were enrolled, there was a limitation in representing Korean mothers. Therefore, more studies are needed to recruit mothers considering their demographic, socio-economic, and cultural characteristics that are representative of mothers in Korea. In addition, a study with large sample size is needed to confirm the factors associated with maternal fatigue in Korea. Second, the quality of interaction in the marital relationship was a factor significantly associated with maternal fatigue, but the current study assessed only whether the spouse offered support in housework and parenting. Further research is needed to determine the relationship between the quality of couples’ interaction and maternal fatigue. Third, children’s sleep shows different patterns depending on the developmental stage. Further research is needed to understand the mother’s fatigue according to the developmental stage of the child. Fourth, sleep quality was retrospectively measured using a self-reported questionnaire, which might have caused self-report bias. To increase the objectivity of sleep measurement, recent studies have measured sleep quality using actigraph devices, a non-invasive method of monitoring human rest and activity cycles. Future research using objective indicators that accurately measure sleep quality and quantity is recommended.

## 5. Conclusions

The current study found that lack of adequate free time in mothers, advanced maternal age, being a housewife, having a moderate income, and frequent night wakings of the child significantly increased fatigue among mothers in the third quantile of fatigue. This finding will assist in attaining better long-term health outcomes such as improved mother-child interaction and better mental health among mothers, thereby contributing to the reduction of fatigue during and beyond the early postpartum period.

## Figures and Tables

**Table 1:** General Characteristics (*n* = 123).

Variables	Categories	Mean ± SD (Range)	*n* (%)
**Maternal characteristics**			
Fatigue score (possible range: 1–7)		4.84 ± 1.08 (1.78–6.89)	
	25% Percentile	3.33 ± 0.72	
	50% Percentile	4.92 ± 0.46	
	75% Percentile	6.16 ± 0.31	
Sleep-wake Pattern			
Sleep quality score (possible range: 0–21)		8.75 ± 3.32 (2–17)	
Psychological pattern			
Parenting stress		93.81 ± 21.13 (40–151)	
(possible range: 0–100)	Normal (0–84)		71 (57.71)
	Slightly high (85–90)		14 (11.38)
	High (91–95)		14 (11.48)
	Very high (≥96)		24 (19.51)
Activity-rest pattern			
Mother’s free time		85.98 ± 115.32 (0–480)	
Individual pattern			
Maternal age (year)		32.11 ± 3.80 (22–42)	
Family type (Living together with)	Child only		1 (0.81)
	Spouse/child		113 (91.87)
	Three generations		9 (7.32)
Working status	No		51 (41.46)
	Yes		72 (58.54)
Education level	High school graduate		15 (12.20)
	College graduate		93 (75.61)
	≥Graduate school		15 (12.20)
Economic status (USD/month)	Low (<2700)		67 (54.47)
	Middle (≥2700, <5000)		48 (39.02)
	High (≥5000)		8 (6.50)
**Child characteristics**			
Age (month)		20.81 ± 9.54 (3–36)	
Environmental pattern			
Number of night wakings	0		20 (16.26)
	1		42 (34.15)
	2		28 (22.76)
	≥3		33 (26.83)

**Table 2:** The Results of Quantile Regression of Factors Associated with Maternal Fatigue (*n* = 123).

Categories	Variables	Categories	OLS (*n* = 123)			QR 0.25 (*n* = 28)			QR 0.50 (*n* = 67)			QR 0.75 (*n* = 28)		
			β (SE)		*p*	β (SE)		*p*	β (SE)		*p*	β (SE)		*p*
Sleep-wake pattern	Maternal sleep quality		0.14	(0.03)	<0.0001	0.21	(0.03)	<0.0001	0.13	(0.03)	<0.0001	0.05	(0.02)	0.005
Psychological pattern	Parenting stress	Normal												
		Slightly high	0.47	(0.28)	0.098	0.42	(0.32)	0.193	0.32	(0.26)	0.210	0.33	(0.18)	0.069
		High	0.20	(0.29)	0.489	0.07	(0.33)	0.836	0.13	(0.27)	0.618	0.08	(0.19)	0.655
		Very high	0.71	(0.24)	0.004	0.70	(0.27)	0.011	0.73	(0.22)	0.001	0.59	(0.15)	0.000
Activity-rest pattern	Mother’s free time		−0.001	(0.001)	0.353	0.000	(0.001)	0.808	−0.002	(0.001)	0.027	−0.002	(0.001)	0.004
Individual pattern	Age (year)		0.03	(0.02)	0.234	0.01	(0.03)	0.614	0.03	(0.02)	0.122	0.04	(0.02)	0.017
	Working status	No												
		Yes	0.02	(0.19)	0.933	0.21	(0.21)	0.327	−0.07	(0.17)	0.687	−0.36	(0.12)	0.003
	Education level	High school												
		College graduation	−0.23	(0.31)	0.456	−0.89	(0.35)	0.012	−0.24	(0.28)	0.386	0.17	(0.19)	0.380
		Graduate school	−0.31	(0.40)	0.436	−1.13	(0.45)	0.013	−0.49	(0.36)	0.178	−0.47	(0.25)	0.063
	Economic status (USD/ month)	Low (<2700)												
		Middle (≥2700, <5000)	0.07	(0.19)	0.728	0.21	(0.21)	0.334	0.09	(0.17)	0.592	0.27	(0.12)	0.025
		High (≥5000)	−0.56	(0.37)	0.128	−0.43	(0.41)	0.299	−0.91	(0.33)	0.007	−0.84	(0.23)	0.000
Environmental pattern	Number of night wakings	0												
		1	0.11	(0.27)	0.675	0.15	(0.31)	0.169	−0.04	(0.25)	0.863	0.04	(0.17)	0.831
		2	0.02	(0.29)	0.953	−0.40	(0.33)	0.229	−0.07	(0.26)	0.778	0.21	(0.18)	0.248
		≥3	0.13	(0.29)	0.653	−0.24	(0.32)	0.470	0.06	(0.26)	0.831	0.43	(0.18)	0.022

## Data Availability

The datasets used and/or analysed during the current study are available from the corresponding author on reasonable request.
